# Marjolin’s Ulcer in Patients With Anogenital Lichen Planus: A Systematic Review

**DOI:** 10.1177/12034754251364858

**Published:** 2025-10-31

**Authors:** Miranda K. Branyiczky, Megan S. Lowe, Mohannad Abu-Hilal, Ivan V. Litvinov

**Affiliations:** 1 Michael G. DeGroote School of Medicine, McMaster University, Hamilton, ON, Canada; 2 Queen’s School of Medicine, Queen’s University, Kingston, ON, Canada; 3 Division of Dermatology, McMaster University, Hamilton, ON, Canada; 4 Division of Dermatology, Department of Medicine, McGill University, Montreal, QC, Canada

**Keywords:** Marjolin’s ulcer, lichen planus, erosive lichen planus, squamous cell carcinoma, human papillomavirus

## Abstract

Anogenital lichen planus (ALP) is a chronic inflammatory condition associated with significant morbidity and a potential risk of cutaneous squamous cell carcinoma (cSCC), specifically Marjolin ulcer (MU), a rare, but aggressive, life-threatening complication. This systematic review aimed to describe the clinical characteristics of patients developing MU within ALP and its treatment outcomes. MEDLINE and Embase (inception—November 2024) were searched, identifying studies reporting MU or squamous dysplasia arising within ALP lesions. Thirty-two studies (n = 176 cases) were analyzed, including cohort studies, case series, and reports, and a descriptive analysis was performed. Patients had a mean age of 63.9 years, with 77.3% female. ALP lesions were most commonly vulval (72.2%) in females and penile (22.2%) in males. MU or cSCC precursor prevalence in ALP cohorts ranged from 0.9% to 4.2%. Human papillomavirus was rarely detected (8.4%), suggesting other mechanisms underlying MU development. The mean ALP duration prior to squamous dysplasia was 47.5 months. Metastases were present in 45.7% of cases that reported this outcome. MU treatment primarily involved surgical excision, with recurrence in 32.9% of cases and mortality in 21.1%. Poor disease control, erosive morphology, and long-standing lesions were associated with higher malignancy risk. Overall, this review highlights the malignant potential of ALP and underscores the importance of early detection and multidisciplinary care. Routine anogenital examinations, timely biopsies of suspicious lesions, and aggressive management of ALP may improve outcomes. Further research is needed to elucidate the mechanisms underlying MU development and optimize treatment strategies for this rare but morbid complication.

## Introduction

Lichen planus (LP) is a chronic T-cell mediated skin disease of unknown etiology. It is thought to occur through nonspecific mechanisms such as mast cell degranulation and protease activation that lead to the accumulation of and cytotoxicity by CD4+ and CD8+ T-cells.^1^ LP typically affects those with a genetic predisposition, while contributing factors may include stress, autoimmune disease, local or systemic viral infection (ie, Herpes zoster, Hepatitis C), contact allergy, or a site of previous trauma.^2^

Anogenital LP (ALP) has widely variable morphology, ranging from purple, pruritic papules, and plaques to painful treatment-resistant erosions. The prevalence of ALP is likely underestimated due to lack of routine genital examination by health care providers and the potentially asymptomatic nature of lesions. It has been estimated that 1% of the population is affected by oral LP (OLP), with 20% to 25% of affected women having vulvovaginal disease and 3.6% of men experiencing penile disease.^3^

ALP can lead to persistent symptoms, architectural changes, and morbidity, significantly reducing quality of life and sexual function. Cutaneous squamous cell carcinoma (cSCC) or its precursors have been reported as rare and potentially life-threatening complications of ALP, primarily occurring in severe or long-standing disease. Considering that these cSCCs form in the setting of chronic inflammation and scarring, they should more appropriately be classified as Marjolin ulcers (MU), an cSCC subset that is associated with significantly worse prognosis than other cSCC.^4^ cSCC precursors include “undifferentiated” intraepithelial neoplasia (IN), such as vulvar IN (VIN) and penile IN (PeIN), ~50% of which arise from transforming infections with human papillomavirus (HPV).^5^ In contrast, “differentiated” IN (ie, d-VIN or d-PeIN) are unrelated to HPV and are typically associated with conditions such as long-standing lichen sclerosus or LP; these lesions often progress more rapidly to malignancy.^5^

The estimated prevalence of MU in ALP ranges significantly and in many patients, diagnosis of ALP is not established until the occurrence of MU. It is essential to understand the characteristics, potential risk factors, and outcomes linked to the development of MU in ALP lesions for prompt diagnosis and effective management. In this study, we conducted a systematic review of case reports, case series, and cohort studies published to characterize ALP-associated MU.

## Materials and Methods

A search was conducted in MEDLINE and Embase from inception to November 2024 in keeping with the Preferred Reporting Items for Systematic reviews and Meta-Analyses (PRISMA) guidelines. Variations of the keywords “lichen planus,” “genital,” “squamous cell carcinoma,” and “Marjolin ulcer” were used (Supplemental Table 1) identifying 984 records, after the removal of duplicates. Titles, abstracts, and full texts of retrieved articles were screened by two independent reviewers (M.K.B. and M.S.L.), and conflicts were resolved with mutual discussion (Supplemental Figure 1). A further 4 articles were identified by reviewing citations of retrieved articles. English articles in which adult patients (≥18 years) had developed MU or SCC precursor within an ALP lesion were included. Exclusion criteria included development of MU or precursors in a non-LP affected region, MU development at other mucosal sites, and unpublished conference abstracts. Following screening, 32 articles were included in the final analysis, comprising 1 prospective cohort study, 18 retrospective cohort studies, 2 case series, and 11 case reports. Quality appraisal was performed using the Joanna Briggs Institute Critical Appraisal Tool (Supplemental Tables 2–4). No studies were excluded from the analysis based on quality assessment outcomes.

A descriptive analysis was performed to synthesize the findings of this literature review, given the significant heterogeneity among the included studies. ALP-induced MU prevalence was calculated in cohort studies describing populations with ALP.

## Results

### Patient Characteristics

A total of 176 cases met inclusion criteria, and their characteristics are summarized in Table 1. Patients had a mean age of 63.9 years (range: 23-90 years), and females accounted for 77.3% of cases (n = 136/176). ALP was diagnosed histologically in 98.3% (n = 117/119) of patients that reported a diagnostic method, while only 2 patients had a solely clinical diagnosis. ALP was located in the vulval (72.2%, n = 127/176), vaginal (8.0%, n = 14/176), perianal/anal (1.7%, n = 3/176), perineal (1.7%, n = 3/176), penile (22.2%, n = 39/176), and scrotal (0.6%, n = 1/176) regions. Multiple clinical types of LP aside from the classic presentation (papules and plaques) were observed including erosive (54.4%, n = 43/79) and hypertrophic (16.4%, n = 13/79) subtypes. Many patients reported symptoms of anogenital pruritus, discomfort, pain, erythema, and dyspareunia, often lasting for years prior to diagnosis.

**Table 1.** Demographic and Clinical Characteristics of Patients With ALP and cSCC.

Characteristic	Number (n)	%
Total	176	100
Sex (female; n = 176)	136	77.3
Age (years, mean ± SD)	63.9 ± 42.3	
ALP duration prior to squamous dysplasia [months (mean ± SD); n = 37]	47.5 ± 75.9	
Histology confirmed ALP diagnosis (n = 119)	117	98.3
LP morphology (n = 79)		
Classic	25	31.6
Erosive	43	54.4
Hypertrophic	13	16.4
Type of squamous dysplasia (n = 176)		
MU/cSCC	123	69.9
d-VIN/d-PeIN	36	20.5
VIN/PeIN	14	8.0
Unspecified SCC in situ	20	11.4
Morphology of MU (n = 35)		
Nodule/plaque	30	85.7
Verrucous	6	17.1
Ulcer	4	11.4
Histological grade of MU (n = 119)		
0 (in situ)	69	57.9
1 (well-differentiated)	4	3.4
1(V) (well-differentiated, verrucous)	2	1.7
2 (moderately differentiated)	40	33.6
3 (poorly differentiated)	2	1.7
HPV status positive (n = 107)	9	8.4
Metastatic status (n = 46)		
Nodal microscopic metastasis	20	43.5%
Distant metastasis	3	6.5%
MU and cSCC precursor treatment (n = 101)		
Surgical excision	88	87.1
Topical therapy	7	6.9
Laser	5	5.0
Radiotherapy	4	4.0
Chemotherapy	2	2.0
Outcome (n = 76)		
Resolution following treatment	32	42.1
Recurrence following treatment	25	32.9
Demise due to MU or its complications	16	21.1

Abbreviations: ALP, anogenital lichen planus; cSCC, cutaneous squamous cell carcinoma; d-PeIN, differentiated penile intraepithelial neoplasia; d-VIN, differentiated vulval intraepithelial neoplasia; HPV, human papillomavirus; LP, lichen planus; MU, Marjolin ulcer; PeIN, penile intraepithelial neoplasia; SD, standard deviation; VIN, vulval intraepithelial neoplasia.

Prior medication use was reported inconsistently and typically only on a population level for cohort studies, making it challenging to understand the prior medication use of individuals who developed cSCC or precursors. Twenty-one cases reported medication history on an individual patient level. Most patients (85.7%, n = 18/21) had previously used topical agents for their ALP including topical corticosteroids, pimecrolimus, or antibiotics. Some (23.8%, n = 5/21) had previously used systemic corticosteroids or systemic immunosuppressive medications, such as methotrexate.

### MU and cSCC Precursor Characteristics

Various forms of squamous dysplasia developed as a result of ALP, including MU (69.9%, n = 123/176), d-VIN/d-PeIN (20.5%, n = 36/176), VIN/PeIN (8.0%, n = 14/176), or unspecified SCC in situ (11.4%, n = 20/176). Of the cases that reported the morphology of MU developing in ALP, the most common was a nodule/plaque (85.7%, n = 30/35), followed by a verrucous lesion (17.1%, n = 6/35) and ulcer (11.4%, n = 4/35). In females, most MU involved the vulval region (85.3%, n = 116/136), encompassing specific structures such as the labia majora, labia minora, and vestibule. Some reports provided more detailed descriptions, identifying involvement of areas such as the vagina (5.1%, n = 7/136), clitoral hood (11.0%, n = 15/136), perianal or anal region (2.2%, n = 3/136), and perineum (1.5%, n = 2/136). In men, the most common sites of MU occurrence were the penis or penile shaft (72.5%, n = 29/40), followed by the glans penis (15.0%, n = 6/40), coronal sulcus/foreskin (15.0%, n = 6/40), and scrotum (2.5%, n = 1/40). HPV testing was performed in 107 patients; however, only 8.4% (n = 9/107) were found to have a positive HPV status. Studies used HPV DNA assay alone (41.1%, n = 44/107) or in combination with p16 staining (57.9%, n = 62/107) to determine HPV status. When histologic grade was reported, most lesions were considered in situ (57.9%, n = 69/119; due to the large number of histologically proven cSCC precursors) or moderately differentiated (33.6%, n = 40/119). The mean duration of LP from diagnosis to MU or cSCC precursor onset was 47 months.

### MU Treatment and Outcomes

Among the patients with available metastasis data, 45.7% (n = 21/46) experienced MU/SCC metastasis. MU was treated by surgical excision in most cases (87.1%, n = 88/101); however, a few patients required radiation treatment (4.0%, n = 4/101) or chemotherapy (2.0%, n = 2/101). Four patients with VIN were treated with topical Imiquimod. MU outcomes were reported in 76 patients. Of these, 42.1% (n = 32/76) remained disease-free following treatment, with no reported recurrences. At least 1 recurrence occurred in 32.9% (n = 25/76) of cases, while death due to disease was reported in 21.1% (n = 16/76) of patients. The average follow-up period was 34.6 months. Of the patients that had a mortality outcome, all were female, with a mean age of 73.1 and diagnosis of cSCC rather than a precursor. The majority of cases had prior erosive ALP (56.3%, n = 9/16) and none had a positive HPV status.

### Prevalence Rates of MU in ALP

The included cohort studies focused on two primary populations: a cohort of patients with ALP, some of whom developed MU or SCC precursor, and a cohort of patients with anogenital cancer, a subset of whom had a prior ALP diagnosis or were diagnosed upon histological examination. Rates of MU development by cohort are summarized in Supplemental Table 5. The provided data from cohort studies following patients with LP (which only included female patients) were utilized to determine the overall rate of new lesion development during the follow-up period. Rates of MU development ranged from 0.9% to 4.2%, while rates of VIN varied from 1.0% to 8.2%.

Studies reporting outcomes of patients with cSCC diagnoses included cohorts of females with vulvovaginal carcinoma or VIN and males with penile or scrotal carcinoma or PeIN. The rates of LP in these anogenital MU cohorts ranged from 3.4% to 13.0%, compared with 1.0% to 2.9% in PeIN cohorts.

## Discussion

cSCC is the second most common type of skin cancer, typically associated with risk factors such as chronic sun exposure, fair skin, and immunosuppression.^6^ MU is a highly aggressive, ulcerating form of cSCC, which develops in chronic wounds or inflammatory lesions. Individuals with chronic ALP lesions may face an increased risk of developing MU, a potentially life-threatening complication. This review highlights the reported characteristics and outcomes of MU and cSCC precursors in patients with ALP.

While the pathogenesis of MU in inflammatory lesions is known to differ from that in sun-damaged areas, the underlying mechanisms remain unclear. It is hypothesized that the immunosuppressive state observed in individuals with LP may contribute to cancer development, aligning with the concept of the “immunocompromised cutaneous district.”^7,8^ This theory suggests that alterations in the local cutaneous immune system may facilitate the emergence of dysimmune reactions, infections, or tumours within the affected site. In general, chronic inflammation plays an important role in tumor development and promotion. Initially, inflammation causes oxidative stress and DNA damage, increasing the production of pro-inflammatory cytokines, such as IL-6 and tumour necrosis factor-α.^9^ Then, inflammatory cells become an important source of tumour-promoting cytokines and growth factors that facilitate oncogenesis.^10^ In the context of chronic wounds, lymphatic vessel dysfunction and resulting lymph statis hinder the normal migration of immune cells, preventing them from counteracting malignant transformation in affected tissues.^11^ The stroma surrounding wounds is rich with growth factors (ie, platelet-derived growth factor and vascular endothelial growth factor), which further support tumour growth.^12^ In LP, long-standing inflammation, tissue destruction (potentially exacerbated by persistent pruritus), and accelerated cellular turnover may disrupt the local cutaneous district and over time predispose to cancer development.^13^ It has also been proposed that local immunosuppression may be related to the underlying immunosuppressive state associated with autoimmune conditions often linked to LP and may be potentiated by the topical treatments commonly employed for the disease.^14^

There remains little reported evidence of molecular mechanisms that underlie the oncogenic transformation of ALP; however, a few studies have examined the inflammatory mediators that may increase oral cavity cancer risk in OLP. One study demonstrated that neutrophils in OLP and oral SCC overexpress B-cell-activating factor, a tumour-promoting molecule that may link the two conditions.^15^ Similar mechanisms may be involved in the tumorigenesis of ALP; however, further research is needed.

HPV-positive MU arising in ALP was also noted in this review. It is worth highlighting that the acquisition HPV may be more prevalent in inflammatory LP lesions, especially those with an erosive morphology.^14^ Inflammation may also trigger reactivation of subclinical infection. As such, despite its low prevalence in our study population, HPV vaccine administration should be considered soon after ALP detection.^16^ While HPV infection is not a prerequisite for squamous malignancy, as demonstrated by the high proportion of HPV-negative cases in this review, its presence may increase the likelihood of dysplastic transformation within some ALP lesions.

The rate of LP-associated anogenital MU observed in our study was comparable with or lower than previously reported rates. A systematic review found that the prevalence of LP in association with vulval cSCC varied widely, ranging from 1% to 33%; however, they noted that there was insufficient study information to determine incidence rate.^17^ In the cohort studies included within this review, the percentage of patients that developed MU or cSCC precursor was narrower, between 0.9% and 4.2%. Of note, the same review also examined the prevalence and incidence of LS in association with cSCC, which was estimated to range from 0% to 83% and 1.16 to 13.67/1000 person-years, respectively, confirming that malignancy associated with LS is more common than with LP.^17^

High-potency topical corticosteroids, such as clobetasol dipropionate or betamethasone dipropionate, remain a cornerstone of ALP treatment.^18^ Despite this, the impact of long-term corticosteroid use on cancer risk is not fully understood.^16^ Studies included in this review often reported the use of systemic immunosuppressive treatments at the cohort level, making it difficult to determine which MU patients had received these therapies. Given the established link between prolonged immunosuppression and increased SCC risk, it would be valuable to determine whether patients with MU had higher exposure to systemic immunosuppressants and which specific medications were associated with MU development.^19^ This lack of granular reporting represents a limitation of our results. Notably, in one study examining a cohort of women with vulval LP, it was noted that all patients who developed d-VIN or vulval MU had long-standing, severe, treatment-resistant disease.^20^ Similarly in case reports of male ALP, MU often occurred following long-standing active or relapsing disease.^21-23^ Therefore, poor disease control may in itself be a risk factor for malignant progression, warranting the treatment of ALP lesions.

Mortality was documented in 21.1% of patients that reported this outcome, while disease recurrence was observed in 32.9% of cases. These findings highlight the importance of aggressive surgical management of malignant lesions, particularly MU, to reduce the risk of recurrence and mortality. Radiation therapy or laser treatments may offer alternative options when surgical management is contraindicated. However, these approaches were infrequently reported in our review, and their efficacy remains uncertain. To prevent disease progression to such advanced stages, comprehensive full-body skin examinations are required. Anogenital regions are known to be commonly overlooked during these examinations, which can contribute to delayed MU diagnosis and a worse prognosis.^24^ Raising awareness of LP’s malignant potential among patients and healthcare providers, alongside multidisciplinary collaboration in dermatology, gynecology, and urology, can enhance earlier detection and improve outcomes for patients with ALP and associated malignancies.

This study included various forms of squamous dysplasia, ranging from SCC in situ (eg, VIN, PeIN) to aggressive, recurrent, and fatal cSCC. In the context of ALP-related squamous dysplasia, cSCC is the most commonly used term in the medical literature, likely due to its frequent discussion in the fields of gynecology, urology, and pathology. However, labelling ALP-associated malignancies as cSCC may minimize their perceived severity.

MU is associated with higher rates of lymph node involvement, distant metastasis, and mortality than cSCC.^25,26^ Additionally, MU has been reported to have a shorter latency period for oncogenesis than UV-related cSCC, indicating a more aggressive tumour pathology.^25^ The time from ALP diagnosis to MU onset varied widely, from a few months to 25 years, suggesting that different disease mechanisms may drive cases with shorter versus longer latencies. In some cases, ALP and MU were diagnosed simultaneously, potentially due to limited disease awareness. Further research is needed to identify which ALP lesions are at higher risk of rapid malignant transformation.

The use of the term “Marjolin’s ulcer” to describe ALP-associated cSCC is supported by the significant recurrence and mortality rates outlined in this review. Adopting this terminology more consistently may better convey the aggressive nature of these malignancies. Therefore, it is the shared responsibility of authors, reviewers, and journal editors to promote the correct usage of this terminology in future research.

Our study is limited by several factors. The studies included were all observational, with heterogeneous reporting of outcomes, which hindered the ability to conduct robust regression analyses and limits the generalizability of findings. Furthermore, the results may be influenced by reporting bias, potentially overrepresenting cases with poorer outcomes.

To better understand the prevalence of MU arising in ALP lesions, its underlying mechanisms and treatment approaches, larger clinical trials are needed. Clinicians should ensure a regular follow-up for all patients with LP to optimize disease management and facilitate early detection of malignancies, especially in those receiving immunosuppressive treatments or presenting other risk factors for MU development. Suspicious lesions should be biopsied to exclude malignancy and facilitate timely intervention.

## Supplemental Material

sj-docx-1-cms-10.1177_12034754251364858 – Supplemental material for Marjolin’s Ulcer in Patients With Anogenital Lichen Planus: A Systematic Review Supplemental material, sj-docx-1-cms-10.1177_12034754251364858 for Marjolin’s Ulcer in Patients With Anogenital Lichen Planus: A Systematic Review by Miranda K. Branyiczky, Megan S. Lowe, Mohannad Abu-Hilal and Ivan V. Litvinov in Journal of Cutaneous Medicine and Surgery
