# Supplementary material for: Marjolin’s Ulcer in Patients With Anogenital Lichen Planus: A Systematic Review
Source: J Cutan Med Surg. 2025 Oct 31;30(3):265–70. doi: 10.1177/12034754251364858 (PMC13216558; doi:10.1177/12034754251364858)
Supplement: sj-docx-1-cms-10.1177_12034754251364858 – Supplemental material for Marjolin’s Ulcer in Patients With Anogenital Lichen Planus: A Systematic Review [file sj-docx-1-cms-10.1177_12034754251364858.docx]

**Supplemental Materials**

**Supplemental Table 1.** Search Strategy for literature screening.

**Embase <1974 to Nov 4, 2024>**

| **#** | **Query** | **Results from Nov 5, 2024** |
| --- | --- | --- |
| 1 | exp lichen planus/ | 15,067 |
| 2 | ("Lichen planus" OR "lichenoid erupt*" OR "lichen ruber" OR “LP”).ti,ab,kw. | 53,948 |
| 3 | exp squamous cell carcinoma/ | 226,816 |
| 4 | (neoplasm* OR carcinoma* OR tumor* OR tumour* OR neoplasia* OR cancer* OR malignan* OR lesion* OR squamous cell ca* OR Marjolin ulcer*).ti,ab,kw. | 6,586,673 |
| 5 | exp genital system/ OR exp genital skin/ OR exp anal canal/ OR exp perineum/ | 781,939 |
| 6 | (Vagina* OR vulva* OR clitoris OR hymen OR labia OR penis OR penile OR foreskin OR urethra* OR scrotum OR scrotal OR testis OR testes OR testic*  OR "reproductive organ*" OR "sexual organ*" OR pudenda* OR genital* OR anogenital* OR anal OR anus  OR perianal*).ti,ab,kw. | 650,890 |
| 7 | 1 OR 2 | 58,634 |
| 8 | 3 OR 4 | 6,599,617 |
| 9 | 5 OR 6 | 1,206,319 |
| 10 | 7 AND 8 AND 9 | 1,107 |
| 11 | limit 10 to (human and english language) | **901** |

**Ovid MEDLINE(R) ALL <1946 to Nov 4, 2024>**

| **#** | **Query** | **Results from Nov 5, 2024** |
| --- | --- | --- |
| 1 | exp lichen planus/ | 9,031 |
| 2 | ("Lichen planus" OR "lichenoid erupt*" OR "lichen ruber" OR “LP”).ti,ab,kf. | 39,504 |
| 3 | exp Carcinoma, Squamous Cell/ | 156,764 |
| 4 | (neoplasm* OR carcinoma* OR tumor* OR tumour* OR neoplasia* OR cancer* OR malignan* OR lesion* OR squamous cell ca* OR Marjolin ulcer*).ti,ab,kf. | 4,957,240 |
| 5 | exp genitalia/ OR  exp anus diseases/ OR exp anus neoplasms/ | 625,097 |
| 6 | (Vagina* OR vulva* OR clitoris OR hymen OR labia OR penis OR penile OR foreskin OR urethra* OR scrotum OR scrotal OR testis OR testes OR testic*  OR "reproductive organ*" OR "sexual organ*" OR pudenda* OR genital* OR anogenital* OR anal OR anus  OR perianal*).ti,ab,kf. | 505,296 |
| 7 | 1 OR 2 | 41,712 |
| 8 | 3 OR 4 | 4,967,324 |
| 9 | 5 OR 6 | 940,656 |
| 10 | 7 AND 8 AND 9 | 535 |
| 11 | limit 10 to (human and english language) | 378 |


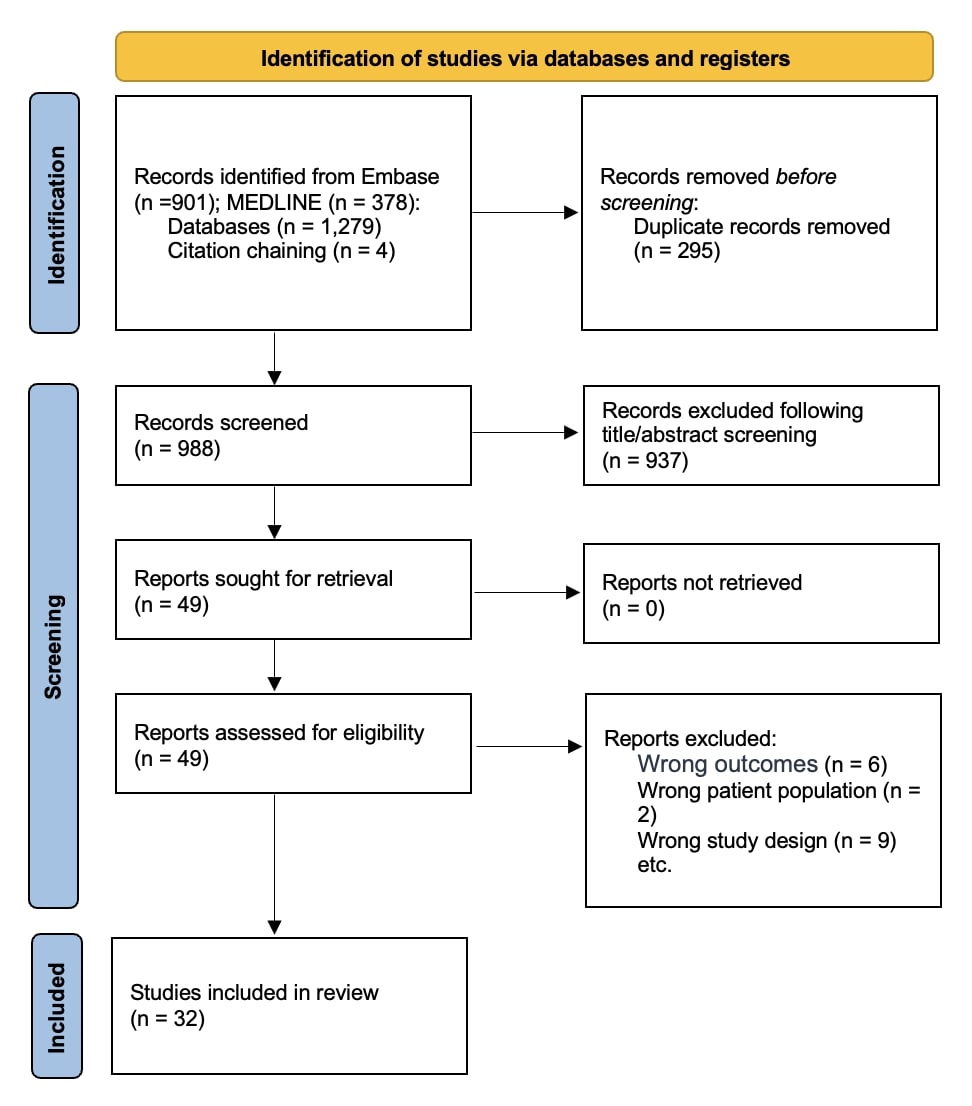


**Supplemental Figure 1.** Identification of studies from databases using Systematic Reviews and Meta-Analyses (PRSMA) guidelines. Figure adapted from http://prisma-statement.org

**Supplemental Table 2.** Quality appraisal of case reports using the Joanna Briggs Institute Critical Appraisal Tool for case reports.

| **Study** | **Were patient’s demographic characteristics clearly described?** | **Was the patient’s history clearly described and presented as a timeline?** | **Was the current clinical condition of the patient on presentation clearly described?** | **Were diagnostic tests or assessment methods and the results clearly described?** | **Was the intervention(s) or treatment procedure(s) clearly described?** | **Was the post-intervention clinical condition clearly described?** | **Were adverse events (harms) or unanticipated events identified and described?** | **Does the case report provide takeaway lessons?** | **Overall Appraisal*** |
| --- | --- | --- | --- | --- | --- | --- | --- | --- | --- |
| Dwyer et al., 1995 | Y | Y | Y | Y | Y | Y | N | Y | Good |
| Leal-Khouri et al., 1994 | Y | Y | Y | Y | Y | Y | N | N | Good |
| Fundaro et al., 1998 | Y | Y | Y | Y | Y | Y | N | Y | Good |
| Jones et al., 2001 | Y | Y | Y | Y | Y | Y | Y | Y | Good |
| Tjalma et al., 2017 | Y | Y | Y | Y | Y | Y | N | Y | Good |
| Franck et al., 1995 | Y | Y | Y | Y | Y | Y | N | Y | Good |
| Cox et al., 1996 | Y | Y | Y | Y | Y | Y | Y | Y | Good |
| Hoshi et al., 2008 | Y | Y | Y | Y | Y | Y | N | Y | Good |
| DeOliveiraLeal et al., 2020 | Y | Y | Y | Y | Y | N | N | Y | Good |
| Chiu et al., 2011 | Y | Y | Y | Y | Y | Y | Y | Y | Good |
| Ruocco et al., 1989 | Y | Y | Y | Y | Y | Y | N | Y | Good |
| *Good quality: studies answering “Yes” to 6 to 8 questions; Moderate quality: “Yes” to 3-5 questions; Poor quality: “Yes”: to 0-2 questions  N: no; Y: yes | | | | | | | | | |

**Supplemental Table 3:** Quality appraisal of case series using the Joanna Briggs Institute Critical Appraisal Tool for case series.

| **Study** | **Were there clear criteria for inclusion in the case series?** | **Was the condition measured in a standard, reliable way for all participants included in the case series?** | **Were valid methods used for identification of the condition for all participants included in the case series?** | **Did the case series have consecutive inclusion of participants?** | **Did the case series have complete inclusion of participants?** | **Was there clear reporting of the demographics of the participants in the study?** | **Was there clear reporting of clinical information of the participants?** | **Were the outcomes or follow up results of cases clearly reported?** | **Was there clear reporting of the presenting site(s)/clinic(s) demographic information?** | **Was statistical analysis appropriate?** | **Overall Appraisal*** |
| --- | --- | --- | --- | --- | --- | --- | --- | --- | --- | --- | --- |
| Lewis et al., 1994 | Y | Y | Y | N | Y | Y | Y | Y | N | N | Good |
| Bain et al., 1989 | N | Y | Y | N | Y | Y | Y | Y | N | N | Moderate |
| *Good quality: studies answering “Yes” to 7 to 10 questions; Moderate quality: “Yes” to 3-6 questions; Poor quality: “Yes”: to 0-2 questions  N: no; Y: yes | | | | | | | | | | | |

**Supplemental Table 4.** Quality appraisal of cohort studies using the Joanna Briggs Institute Critical Appraisal Tool for cohort studies.

| **Study** | **Were the two groups similar and recruited from the same population?** | **Were the exposures measured similarly to assign people**  **to both exposed and unexposed groups?** | **Was the exposure measured in a valid and reliable way?** | **Were confounding factors identified?** | **Were strategies to deal with confounding factors stated?** | **Were the groups/participants free of the outcome at the start of the study (or at the moment of exposure)?** | **Were the outcomes measured in a valid and reliable way?** | **Was the follow up time reported and sufficient to be long enough for outcomes to occur?** | **Was follow up complete, and if not, were the reasons to loss to follow up described and explored?** | **Were strategies to address incomplete follow up utilized?** | **Was appropriate statistical analysis used?** | **Overall Appraisal*** |
| --- | --- | --- | --- | --- | --- | --- | --- | --- | --- | --- | --- | --- |
| Kristiansen et al., 2019 | Y | Y | Y | Y | Y | Y | Y | Y | Y | Y | Y | Good |
| Boch et al., 2021 | Y | Y | Y | Y | Y | Y | Y | Y | Y | N | Y | Good |
| Lyra et al., 2021 | Y | N | Y | N | N | Y | Y | Y | N | N | Y | Moderate |
| Santegoets et al., 2010 | N/A | N/A | Y | N | N | Y | Y | Y | Y | N | Y | Moderate |
| Mannweiler et al., 2011 | Y | Y | Y | N | N | N/A | Y | N/A | N/A | N/A | Y | Moderate |
| Cooper et al., 2006 | N/A | N/A | Y | N | N | Y | Y | Y | N | N | Y | Moderate |
| Mannweiler et al., 2013 | Y | Y | Y | N | N | N/A | Y | N/A | N/A | N/A | Y | Moderate |
| Regauer et al., 2016 | N | N | Y | Y | N | Y | Y | Y | Y | N | Y | Moderate |
| Regauer et al., 2014 | N | N | Y | Y | N | Y | Y | Y | Y | N | Y | Moderate |
| Zaki et al., 1996 | N | N | Y | Y | N | N | Y | Y | N | N | Y | Moderate |
| Kirtschig et al., 2005 | Y | N | Y | Y | N | N | Y | Y | N | N | Y | Moderate |
| Simpson et al., 2012 | Y | N | Y | Y | N | N | Y | Y | N | N | Y | Moderate |
| Matoso et al., 2014 | Y | N | Y | N | N | N | Y | Y | N | N | Y | Moderate |
| Derrick et al., 2000 | Y | N | Y | Y | N | N | Y | Y | N | N | Y | Moderate |
| Fahy et al., 2017 | Y | N | Y | N | N | Y | Y | Y | Y | N | Y | Moderate |
| Kherlopian et al., 2020 | Y | N | Y | Y | Y | N | Y | Y | N | N | Y | Moderate |
| Halonen et al., 2018 | Y | Y | Y | N | N | Y | Y | Y | Y | N | Y | Good |
| Porter et al., 2002 | Y | N | Y | Y | Y | N | Y | Y | N | N | Y | Moderate |
| Kennedy et al., 2008 | Y | Y | Y | N | N | Y | Y | Y | Y | Y | Y | Good |
| *Good quality: studies answering “Yes” to 8-11 questions; Moderate quality: “Yes” to 4-7 questions; Poor quality: “Yes” to 0-3 questions  N: no; N/A: Not applicable; Y: yes | | | | | | | | | | | | |

**Supplemental Table 5.** Rates of anogenital lichen planus-associated Marjolin’s Ulcer and squamous dysplasia in cohort studies.

| First author, year | Cohort study type | Cohort disease | Sample size (n) | Type of lesion | Rate of LP associated squamous dysplasia % (n) |
| --- | --- | --- | --- | --- | --- |
| Kherlopian et al., 2020 | Retrospective | Vulval LP | 105 | MU | 1.9% (n=2) |
|  |  |  |  | dVIN | 0.9% (n=1) |
| Boch et al., 2021 | Retrospective | Vulval LP | 24 | MU | 4.2% (n=1) |
|  |  |  |  | VIN | 8.2% (n=2) |
| Regauer et al., 2016 | Retrospective | Vulval LP | 584 | MU | 1.7% (n=10) |
| Lyra et al., 2021 | Retrospective | Vulval LP | 127 | MU | 1.6% (n=2) |
| Santgoets et al., 2010 | Retrospective | Vulval LP | 95 | MU | 2.1% (n=2) |
| Cooper et al., 2006 | Prospective | Erosive vulval LP | 114 | MU | 1.8% (n=2) |
|  |  |  |  | VIN | 6.1% (n=7) |
| Kirtschig et al., 2005 | Retrospective | Vulval LP | 44 | MU | 2.3% (n=1) |
| Simpson et al., 2012 | Retrospective | Erosive vulval LP | 172 | MU | 1.2% (n=2) |
|  |  |  |  | VIN | 1.2% (n=2) |
| Kennedy et al., 2008 | Retrospective | Erosive vulval LP | 113 | MU | 0.9% (n=1) |
| Fahy et al., 2017 | Retrospective | Vulval LP | 100 | MU | 2.0% (n=2) |
|  |  |  |  | VIN | 1.0% (n=1) |
| Halonen et al., 2018 | Retrospective | Females with LP | 13,100 | MU | 0.1% (n=18) |
| Matoso et al., 2014 | Retrospective | Scrotal cancer | 29 | MU | 3.4% (n=1) |
| Porter et al., 2002 | Retrospective | PeIN | 35 | MU | 2.9% (n=1) |
| Derrick et al., 2000 | Retrospective | Females with anogenital carcinoma | 23 | MU | 13.0% (n=3) |
| Mannweiler et al., 2013 | Retrospective | Penile SCC | 123 | MU | 10.6% (n=13) |
| Mannweiler et al., 2011 | Retrospective | Penile cancer | 164 | MU | 4.3% (n=7) |
|  |  |  |  | d-PeIN | 4.9% (n=8) |
| Zaki et al., 1996 | Retrospective | Vulval cancer | 50 | MU | 6.0% (n=3) |
| Kristiansen et al., 2019 | Retrospective | PeIN | 580 | MU | 1.0% (n=1) |

Abbreviations: LP, lichen planus; MU, Marjolin’s Ulcer; PeIN, penile intraepithelial neoplasia; VIN, vulval intraepithelial neoplasia.

**Supplemental Appendix 1.** Complete list of references for included studies.

Bain L, Geronemus R. The association of lichen planus of the penis with squamous cell carcinoma in situ and with verrucous squamous carcinoma. *J Dermatol Surg Oncol.* 1989;15(4):413-417. doi:10.1111/j.1524-4725.1989.tb03247.x

Boch K, Langan EA, Zillikens D, Ludwig RJ, Kridin K. Retrospective analysis of the clinical characteristics and patient-reported outcomes in vulval lichen planus: Results from a single-center study. *J Dermatol.* 2021;48(12):1913-1917. doi:10.1111/1346-8138.16191

Chiu TL, Jones RW. Multifocal multicentric squamous cell carcinomas arising in vulvovaginal lichen planus. *J Low Genit Tract Dis.* 2011;15(3):246-247. doi:10.1097/LGT.0b013e31820bad90

Cooper SM, Wojnarowska F. Influence of treatment of erosive lichen planus of the vulva on its prognosis. *Arch Dermatol*. 2006;142(3):289-294. doi:10.1001/archderm.142.3.289

Cox NH. Squamous cell carcinoma arising in lichen planus of the penis during topical cyclosporin therapy. *Clin Exp Dermatol.* 1996;21(4):323-324. doi:10.1111/j.1365-2230.1996.tb00112.x

de Oliveira Leal ML, Alencar LRPJ, Santana SC, et al. Penile squamous cell carcinoma and lichen planus. *Surg Exp Pathol*. 2020;3(1).

Derrick EK, Ridley CM, Kobza-Black A, McKee PH, Neill SM. A clinical study of 23 cases of female anogenital carcinoma. *Br J Dermatol.* 2000;143(6):1217-1223. doi:10.1046/j.1365-2133.2000.03891.x

Dwyer CM, Kerr RE, Millan DW. Squamous carcinoma following lichen planus of the vulva. *Clin Exp Dermatol.* 1995;20(2):171-172. doi:10.1111/j.1365-2230.1995.tb02677.x

Fahy CMR, Torgerson RR, Davis MDP. Lichen planus affecting the female genitalia: A retrospective review of patients at Mayo Clinic. *J Am Acad Dermatol*. 2017;77(6):1053-1059. doi:10.1016/j.jaad.2017.07.030

Franck JM, Young AW Jr. Squamous cell carcinoma in situ arising within lichen planus of the vulva. *Dermatol Surg*. 1995;21(10):890-894. doi:10.1111/j.1524-4725.1995.tb00718.x

Fundarò S, Spallanzani A, Ricchi E, et al. Squamous-cell carcinoma developing within anal lichen planus: report of a case. *Dis Colon Rectum*. 1998;41(1):111-114. doi:10.1007/BF02236905

Halonen P, Jakobsson M, Heikinheimo O, Riska A, Gissler M, Pukkala E. Cancer risk of Lichen planus: A cohort study of 13,100 women in Finland. *Int J Cancer.* 2018;142(1):18-22. doi:10.1002/ijc.31025

Hoshi A, Usui Y, Terachi T. Penile carcinoma originating from lichen planus on glans penis. *Urology*. 2008;71(5):816-817. doi:10.1016/j.urology.2008.01.038

Jones RW, Rowan DM, Kirker J, Wilkinson EJ. Vulval lichen planus: progression of pseudoepitheliomatous hyperplasia to invasive vulval carcinomas. *BJOG.* 2001;108(6):665-666. doi:10.1111/j.1471-0528.2001.00134.x

Kennedy CM, Peterson LB, Galask RP. Erosive vulvar lichen planus: a cohort at risk for cancer?. *J Reprod Med.* 2008;53(10):781-784.

Kherlopian A, Fischer G. Vulvar malignancy in biopsy-proven vulval lichen planus: A retrospective review of 105 cases. *Australas J Dermatol.* 2020;61(4):386-388. doi:10.1111/ajd.13368

Kirtschig G, Wakelin SH, Wojnarowska F. Mucosal vulval lichen planus: outcome, clinical and laboratory features [published correction appears in J Eur Acad Dermatol Venereol. 2005 Jul;19(4):530]. *J Eur Acad Dermatol Venereol*. 2005;19(3):301-307. doi:10.1111/j.1468-3083.2004.01167.x

Kristiansen S, Svensson Å, Drevin L, Forslund O, Torbrand C, Bjartling C. Risk Factors for Penile Intraepithelial Neoplasia: A Population-based Register Study in Sweden, 2000-2012. *Acta Derm Venereol*. 2019;99(3):315-320. doi:10.2340/00015555-3083

Leal-Khouri S, Hruza GJ. Squamous cell carcinoma developing within lichen planus of the penis. Treatment with Mohs micrographic surgery. *J Dermatol Surg Oncol.* 1994;20(4):272-276. doi:10.1111/j.1524-4725.1994.tb01624.x

Lewis FM, Harrington CI. Squamous cell carcinoma arising in vulval lichen planus. *Br J Dermatol.* 1994;131(5):703-705. doi:10.1111/j.1365-2133.1994.tb04987.x

Lyra J, Melo C, Figueiredo R, et al. Erosive Vulvar Lichen Planus and Risk of Vulvar Neoplasia. *J Low Genit Tract Dis.* 2021;25(1):71-75. doi:10.1097/LGT.0000000000000581

Mannweiler S, Sygulla S, Beham-Schmid C, Razmara Y, Pummer K, Regauer S. Penile carcinogenesis in a low-incidence area: a clinicopathologic and molecular analysis of 115 invasive carcinomas with special emphasis on chronic inflammatory skin diseases. *Am J Surg Pathol*. 2011;35(7):998-1006. doi:10.1097/PAS.0b013e3182147e59

Mannweiler S, Sygulla S, Winter E, Regauer S. Two major pathways of penile carcinogenesis: HPV-induced penile cancers overexpress p16ink4a, HPV-negative cancers associated with dermatoses express p53, but lack p16ink4a overexpression. *J Am Acad Dermatol.* 2013;69(1):73-81. doi:10.1016/j.jaad.2012.12.973

Matoso A, Ross HM, Chen S, Allbritton J, Epstein JI. Squamous neoplasia of the scrotum: a series of 29 cases. *Am J Surg Pathol.* 2014;38(7):973-981. doi:10.1097/PAS.0000000000000192

Porter WM, Francis N, Hawkins D, Dinneen M, Bunker CB. Penile intraepithelial neoplasia: clinical spectrum and treatment of 35 cases. *Br J Dermatol.* 2002;147(6):1159-1165. doi:10.1046/j.1365-2133.2002.05019.x

Regauer S, Eberz B, Reich O. Human Papillomavirus-Induced Squamous Intraepithelial Lesions in Vulvar Lichen Planus. *J Low Genit Tract Dis.* 2016;20(4):360-364. doi:10.1097/LGT.0000000000000248

Regauer S, Reich O, Eberz B. Vulvar cancers in women with vulvar lichen planus: a clinicopathological study. *J Am Acad Dermatol.* 2014;71(4):698-707. doi:10.1016/j.jaad.2014.05.057

Ruocco V, Satriano RA, De Rosa G, Pettinato G, Gombos F. Malignancy in lichen planus. *Int J Dermatol*. 1989;28(8):542-544. doi:10.1111/j.1365-4362.1989.tb04612.x

Santegoets LA, Helmerhorst TJ, van der Meijden WI. A retrospective study of 95 women with a clinical diagnosis of genital lichen planus. *J Low Genit Tract Dis*. 2010;14(4):323-328. doi:10.1097/LGT.0b013e3181d73622

Simpson RC, Littlewood SM, Cooper SM, et al. Real-life experience of managing vulval erosive lichen planus: a case-based review and U.K. multicentre case note audit. *Br J Dermatol.* 2012;167(1):85-91. doi:10.1111/j.1365-2133.2012.10919.x

Tjalma WA, Siozopoulou V, Huizing MT. A clitoral verrucous carcinoma in an area of lichen planus has aggressive features. *World J Surg Oncol*. 2017;15(1):7. Published 2017 Jan 6. doi:10.1186/s12957-016-1069-0

Zaki I, Dalziel KL, Solomonsz FA, Stevens A. The under-reporting of skin disease in association with squamous cell carcinoma of the vulva. *Clin Exp Dermatol*. 1996;21(5):334-337.
